# The multiple roles of phosphate in muscle fatigue

**DOI:** 10.3389/fphys.2012.00463

**Published:** 2012-12-11

**Authors:** David G. Allen, Sofie Trajanovska

**Affiliations:** School of Medical Sciences, Bosch Institute and Sydney School of Medicine, University of SydneySydney, NSW, Australia

**Keywords:** skeletal muscle, fatigue, intracellular calcium, inorganic phosphate, myofibrillar performance

## Abstract

Muscle fatigue is the decline in performance of muscles observed during periods of intense activity. ATP consumption exceeds production during intense activity and there are multiple changes in intracellular metabolites which may contribute to the changes in crossbridge activity. It is also well-established that a reduction in activation, either through action potential changes or reduction in Ca^2+^ release from the sarcoplasmic reticulum (SR), makes an additional contribution to fatigue. In this review we focus on the role of intracellular inorganic phosphate (P_i_) whose concentration can increase rapidly from around 5–30 mM during intense fatigue. Studies from skinned muscle fibers show that these changes substantially impair myofibrillar performance although the effects are strongly temperature dependent. Increased P_i_ can also cause reduced Ca^2+^ release from the SR and may therefore contribute to the reduced activation. In a recent study, we have measured both P_i_ and Ca^2+^ release in a blood-perfused mammalian preparation and the results from this preparation allows us to test the extent to which the combined effects of P_i_ and Ca^2+^ changes may contribute to fatigue.

## Introduction

Muscle fatigue can be defined as the decline of muscle performance associated with intense muscle activity. Many mechanisms contribute to this decline, from psychological factors within the cortex down to the state of individual proteins inside the muscle. To study mechanisms in detail it is usually necessary to use a simplified preparation and a well-defined pattern of stimulation. One approach has been to use isolated small preparations of muscle and stimulate with intermittent, maximal, isometric tetani; obviously central (cortical) mechanisms do not occur in this preparation and the effects of changes in blood flow are also absent. Another approach is to use human subjects and induce fatigue by maximal or near-maximal contractions repeated regularly. In suitable muscles both force and metabolite changes can be measured simultaneously and repeatedly. Two limitations to this approach are that both the force and the metabolites are usually averaged across a muscle which consists of various fiber types with different properties. In addition the degree of central vs. peripheral fatigue is often uncertain in human subjects. Given the interpretative difficulties of these two approaches, a third approach is of great importance; application of known metabolite changes to skinned muscle preparations. In this preparation, the metabolites are defined and can be changed at will and the fiber type can be determined. Extensive data is available documenting how the performance of the contractile proteins is affected by individual metabolite changes.

In this short review we will (1) discuss the evidence that inorganic phosphate (P_i_) contributes to the changes in crossbridge behavior, (2) consider the evidence that phosphate contributes to the reduced Ca^2+^ release, (3) examine the correlation between force and P_i_ in fatiguing muscles, and (4) describe a new model of fatigue in which both P_i_ and intracellular tetanic Ca^2+^ concentration ([Ca^2+^]_i_) can be measured and analyse how these two factors in combination contribute to the force decline. This type of analysis involves many assumptions and can only give a general indication of the magnitude of the contribution that might arise from these sources. It is accepted that many other factors, with both positive and negative effects, contributed to the overall effect.

Many metabolites change in fatigue and a number of them have been shown in skinned fibers to affect the performance of the contractile proteins or Ca^2+^ release. Historically pH change has been widely considered a major cause of fatigue acting through a reduction in Ca^2+^ sensitivity (Fabiato and Fabiato, 1978). However, over the last two decades its contribution has been reassessed mainly because the effects of pH on isometric force are much reduced at mammalian body temperatures (Pate et al., 1995). In contrast it seems that acidosis reduces shortening velocity and muscle power at body temperature (Fitts, 2008) so that acidosis may still be of importance in many kinds of fatigue. This reassessment has been documented extensively elsewhere (for review see Westerblad et al., 2002; Cairns, 2006; Cooke, 2007). Other metabolites with probable roles in fatigue include ATP, ADP, PCr, Mg, and reactive oxygen and nitrogen species (Allen et al., 2008b). In addition many proteins display post-translational changes which affect their function, for instance phosphorylation, nitrosylation, and oxidation and the importance of these are starting to emerge (Bellinger et al., 2008; Mollica et al., 2012).

Muscles use ATP as the immediate source of energy but the ADP and P_i_ produced are regenerated by a series of metabolic pathways which utilize stores of PCr, glycogen, and lipid within the cell. Glycogen within the cell can be utilized by anaerobic glycolysis whose net products are lactate and protons. The metabolic pathways that regenerate ATP are turned on rapidly and tightly regulated so that the net effect is that ATP concentration is only marginally reduced by moderate exercise but there are major increases in P_i_, ADP, creatine, and protons while PCr and glycogen decrease. The changes in concentration of ATP, PCr, P_i_, and ADP occur in a relatively stereotyped fashion which has been documented in numerous studies (Dawson et al., 1978; Cady et al., 1989; Lanza et al., 2006). Conversely the degree of acidosis observed is more variable because the rate and extent to which anaerobic glycolysis is turned on is dependent on the fiber type and the nature of the activity. Furthermore, skeletal muscle fibers contain variable amounts of the lactate transporter which reduce any intracellular accumulation of lactate and protons.

## Phosphate contributes to the decline in crossbridge performance

**Figure 1:**
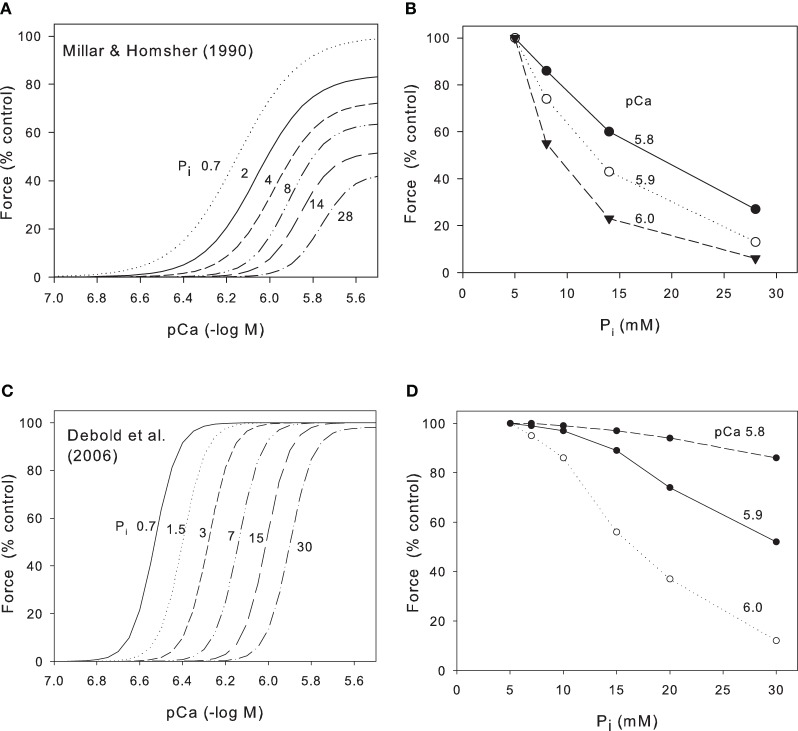
**Effect of P_i_ on relation between pCa and force for skinned skeletal muscle. (A)** Data from rabbit psoas muscle at 10°C (Millar and Homsher, 1990). Ca^2+^ concentrations have been adjusted for the excessively high Ca^2+^ EGTA binding constant used in this study. P_i_ concentrations (mM) as indicated on Panel. Note large fall in maximum Ca^2+^-activated force under these conditions. **(B)** Data from Millar and Homsher replotted to show how force falls as a function of P_i_ at various pCa levels. **(C)** Data from fast gastrocnemius muscle at 30°C (Debold et al., 2006). Published data were at 0.7 mM (no added P_i_) and 30 mM P_i_; we have interpolated curves on the basis that pCa50 is a logarithmic function of P_i_. Note the absence of significant effect of P_i_ on maximum Ca^2+^-activated force under these conditions and that the Debold et al. data is much steeper; Hill coefficients ~8 whereas for Millar and Homsher (1990) Hill coefficients were 2–6. **(D)** Data from Debold et al. (2006) redraw to show force as a function of P_i_ at various pCa levels.

Ruegg et al. (1971), working with skinned insect flight muscle, were the first to note that increased P_i_ inhibited contractile force. This observation was later repeated and extended by many groups working on skinned skeletal muscle (Cooke and Pate, 1985; Millar and Homsher, 1990). These groups demonstrated that, in skinned skeletal muscle at low temperatures (10–20°C), an increase in P_i_ produced a substantial reduction in both maximum Ca^2+^-activated force and in Ca^2+^ sensitivity e.g., Figure 1A from Millar and Homsher (1990). It is generally accepted that this reduction occurs because P_i_ is released from crossbridges at a stage closely associated with force production so that elevation of phosphate accelerates the backward rate of this step and thus reduces force (Takagi et al., 2004; Cooke, 2007; Fitts, 2008). Cooke and Pate and Millar and Homsher showed that both the reduction of force and the shift of Ca^2+^ sensitivity were logarithmic functions of P_i_. Thus, a 10-fold increase in P_i_ produced a reduction in maximum Ca^2+^-activated force to 63% and a reduction of Ca^2+^ sensitivity of 0.2 pCa units (Millar and Homsher, 1990). In order to compare data from skinned fibers with data from intact muscles, we have converted the data in Figure 1A to the relationship between force and P_i_ (Figure 1B). In mammalian muscles the resting myoplasmic [P_i_] is around 5 mM (Kemp et al., 2007; Fitts, 2008) so we have normalized the force to 100% at this [P_i_] and compared the relationship at three different pCa (5.8, 5.9, and 6.0) to represent the levels that may occur in near-maximally activated tetani. Even at high Ca^2+^, the relation between force and P_i_ declines because of the effect of P_i_ on maximum Ca^2+^-activated force and this effect is largest at low P_i_ and decreases as P_i_ increases. At lower Ca^2+^ levels the relationship falls more steeply as the effect of changes in pCa50 (pCa required to give half maximal force) add to the reduced maximum Ca^2+^-activated force.

The early work on skinned muscle cited above was performed at 10–20°C and a major recent development was the discovery that the effects of both pH and P_i_ on skinned fibers are quantitatively and qualitatively different when repeated at near physiological temperatures (Pate et al., 1995; Coupland et al., 2001; Debold et al., 2006). Data from Debold et al. (2006) are shown in Figure 1C who studied fast and slow fibers at both 15°C and 30°C. Over the range of 0.7–30 mM P_i_, maximum Ca^2+^-activated force was reduced by 46% at 15°C but by only 2% at 30°C. In contrast, over the same range of P_i_, the pCa50 was reduced by 0.28 log units at 15°C but was reduced by 0.63 log units at 30°C. Assuming the relation between P_i_ and Ca^2+^ sensitivity is logarithmic (Millar and Homsher, 1990), we have interpolated the data of Debold et al. (2006) at 30°C into a series of force/pCa curves at various P_i_ (Figure 1C). We have then converted this data into the relationship between force and P_i_ for various pCa levels (Figure 1D). Note that in contrast to the data at low temperatures (Figures 1A,B), there is little effect of P_i_ on maximum Ca^2+^-activated force and consequently, at the highest Ca^2+^ (pCa 5.8) P_i_ has relatively little effect on force (Figure 1D). Only when the Ca^2+^ is lower (pCa 5.9 and 6.0) does the force fall substantially and the effect is least at low P_i_ and greater as P_i_ increases.

At low temperatures, increases in both P_i_ and H^+^ depress muscle force and when applied simultaneously the effect is larger and generally assumed to be multiplicative. Nosek et al. (1987) factored out the effect of pH and noted that, after this procedure, the effect on force of a fixed concentration of P_i_ was very variable. However, when the total P_i_ was converted to the H_2_PO^−^_4_ and HPO^2−^_4_ forms, there was a good correlation with the diprotonated form and a poor correlation with the monoprotonated form. On this basis they suggested that it was the diprotonated form of P_i_ which inhibited force and that pH exerted two effects on force; a direct effect of protons and an indirect effect by converting more of the total P_i_ to the diprotonated form. However, some other studies have been unable to reproduce this effect (e.g., Chase and Kushmerick, 1988) and it is generally agreed that this effect is only seen in fast skeletal muscle but not in slow or cardiac muscle. It is also worth noting that the experiments on this topic were at 22°C and it is not known whether the effect persists at mammalian temperatures. Thus, the issue remains unresolved and some experimenters chose to display force data against total P_i_ while others compare force with H_2_PO^−^_4_.

## The effect of P_i_ on Ca^2+^ release

Fatigue is known to involve both reduced crossbridge performance and reduced activation. There are many possible mechanisms by which activation might decline during fatigue (for review see Allen et al., 2008a) but in the present article we focus on the possibility that P_i_ may have some direct effect on activation. This idea was first raised by Fryer et al. (1995) in a study on skinned fibers with intact sarcoplasmic reticulum (SR). Ca^2+^ release was triggered by caffeine and was found to decrease substantially after the preparation had been bathed in a high P_i_ solution. They suggested that during fatigue P_i_ entered the SR reaching concentrations at which it was capable of precipitating with the high levels of Ca^2+^ found in the SR. This Ca^2+^P_i_ precipitate would thus reduce the free Ca^2+^ available in the SR for release (for review see Allen and Westerblad, 2001). This theory was supported by a study in which P_i_ was microinjected into single muscle fibers (Westerblad and Allen, 1996). There was a small reduction in Ca^2+^ sensitivity but a substantial reduction in tetanic Ca^2+^ present after 4 min. These data suggest that much of the P_i_ entered the SR where it precipitated and subsequently reduced Ca^2+^ release. Further support was provided by Laver et al. (2001) who discovered a phosphate permeable channel in the SR which could provide the route of entry for P_i_. This mechanism was strengthened by the observation that the decline in tetanic [Ca^2+^]_i_ which normally occurs during fatigue is reduced or delayed in creatine kinase knockout mice, in which the normal fatigue-induced rise in P_i_ is reduced (Dahlstedt and Westerblad, 2001).

More recently Dutka et al. (2005) have extended earlier work on skinned fibers by showing that the P_i_-induced reduction in Ca release initially observed with caffeine triggered release is also present when Ca release is triggered by T-tubular action potentials. They also showed that the total SR Ca^2+^ was unaffected by P_i_, supporting the idea that Ca^2+^ had precipitated as opposed to leaking out of the SR. In their experiments a 2 min exposure to 30 mM P_i_ was sufficient to reduce Ca^2+^ release to a steady level suggesting that this mechanism would have time to operate during a fatigue protocol of this or greater length. This mechanism appears to be substantially smaller in slow fibers compared to fast, which may contribute to the fatigue-resistance of slow fibers (Posterino and Dunn, 2008).

In summary, a substantial body of evidence points to the possibility that elevated P_i_ contributes to reduced Ca^2+^ release during fatigue. Definitive evidence probably would require measurements of Ca^2+^P_i_ precipitates in the SR and the timecourse of their change during fatigue and recovery. However, the small size of the SR makes this a challenging procedure.

## The correlation between fatigue and [P_i_]

Many studies have correlated the changes in myoplasmic [P_i_] with the changes in force during fatiguing activities. While the measurements of metabolites from isolated muscles and from biopsies of human muscles have been possible for many years, these methods are not easily scaled up to obtaining multiple samples. Thus, a rapid expansion in knowledge occurred with the development of nuclear magnetic resonance which allows non-invasive sampling of metabolites to be repeated frequently. Dawson et al. (1978) pioneered this technique in muscle and were able to plot PCr, Cr, P_i_, ADP, ATP, and H^+^ as functions of the declining force in tetanically stimulated ischaemic frog muscle. H^+^ showed a strong linear correlation with declining force and was therefore a good candidate. P_i_ also correlated well with force decline though at high P_i_ there was some deviation from linearity. This work was performed on frog muscles at 4°C; given the importance of temperature we focus in this section on subsequent studies in humans or mammals at or near body temperature.

**Figure 2:**
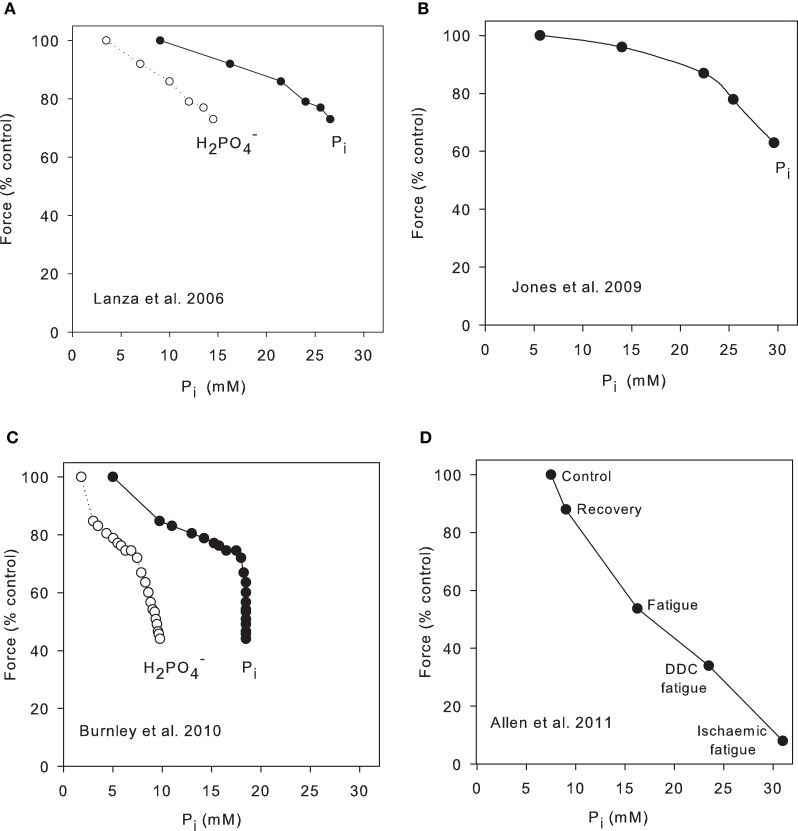
**Correlation between P_i_ and force in intact, fatiguing muscle. (A)** Human subjects performed six 12 s maximal contractions involving dorsiflexion of the ankle and NMR spectra were collected from the tibialis anterior muscle in 12 s periods between contractions (Lanza et al., 2006). In the original publication only H_2_PO^−^_4_ was plotted; we have calculated P_i_ on the basis P_i_ = H_2_PO^−^_4_ (1 + 10^pH−6.75^). **(B)** Stimulated maximal tetani of the human tibialis anterior muscle under ischaemic conditions. 1.6 s tetani with 1.6 s intervals between tetani continued for 32 s (Jones et al., 2009). **(C)** Human subjects performed 60 maximal voluntary contractions of the quadriceps group, each contraction 3 s in duration with 2 s rest between (Burnley et al., 2010). P_i_ and H_2_PO^−^_4_ were recorded between each contraction. Resting P_i_ assumed to be 5 mM. **(D)** Exteriorized mouse tibialis anterior stimulated with maximal tetani of 0.4 s duration repeated every 4 s. Muscles were sampled under control conditions and when fatigue had reached a steady state (12 min). A second protocol involved 0.4 s tetani repeated every 2 s (double duty cycle or DDC fatigue). Some fatigue protocols were recorded after death when circulation had ceased (ischaemic fatigue). Data from Allen et al. (2011).

**Figure 3:**
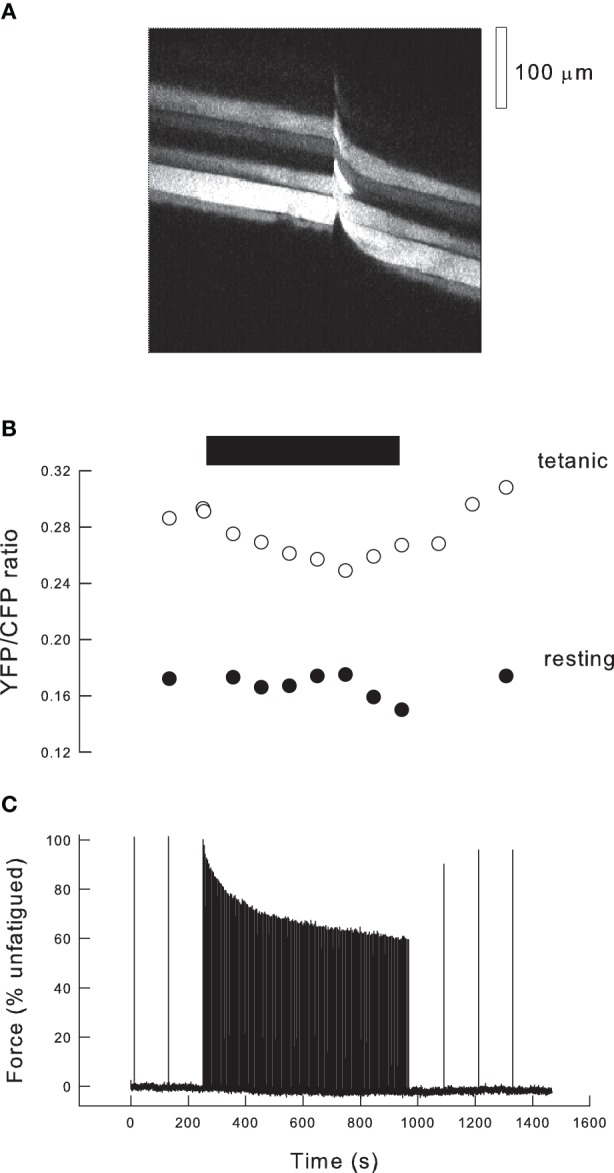
**Fatigue in the exteriorized mouse tibialis anterior muscle. (A)** Fluorescence image of tibialis anterior muscle 10 days after transfection with cameleon (YC2). Image was scanned from L to R over 750 ms, and the muscle stimulated after 400 ms (indicated by movement artifact). Thus, in the L part of the image the fibers are resting whereas in the R part of the image the fibers are tetanized. **(B)** Records of the ratio of fluorescence of YFP/CFP obtained from images such as **(A)** during a fatigue protocol. Black bar indicates period of high duty cycle stimulation. Empty circles indicate ratios determined during tetani (R hand images in Panel **A**), filled circles show ratios from the resting muscle just prior to tetani (L hand images in Panel **A**). **(C)** Record of force during the stimulation protocol. Initially the muscle is stimulated every 2 min and force is stable. When duty cycle is increased to one tetanus every 4 s, force declines exponentially and approaches a steady state. Stimuli are then given every 2 min and force recovers to ~90% of the initial level.

Lanza et al. (2006) examined force and metabolite change in the human tibialis anterior in response to six maximal 12 s contractions separated by 12 s rest periods. In this protocol force declined to 74% control and over this period H_2_PO^−^_4_ increased from 3.5 to 15 mM and showed a near linear relation with force (Figure 2A). We have calculated total P_i_ from their data and this also correlates well with force (Figure 2A). These authors repeated the protocol under ischaemic conditions with similar results. Jones et al. (2009) also studied the human tibialis anterior under ischaemic conditions but using electrical stimulation of the muscle and their data show a comparable relationship between P_i_ and force decline (Figure 2B). ATP consumption was calculated and, after correction for the decline of force, the economy of contraction appeared unchanged during the period of fatigue. A different approach was used by Burnley et al. (2010) who studied the quadriceps group during repeated maximal voluntary contractions, 3 s on 2 s off, continued until the force was stable. P_i_ and H_2_PO^−^_4_ were measured at 5 s intervals throughout, so that the timecourse of P_i_ and force could be followed throughout the procedure. The results show that P_i_ initially changed more quickly than force (Figure 2C) so that the plot has a region where force and P_i_ change reasonably linearly but, because force continued to fall while P_i_ was constant, this gives a near vertical line on the plot. Because pH continued to decline slowly throughout fatigue, the plot of H_2_PO^−^_4_ is more nearly linear over the whole range. Thus, this data supports the idea that pH is making some contribution to fatigue, either directly or through the changing proportions of H_2_PO^−^_4_/total P_i_; alternatively the authors suggest that a central component to fatigue may become more prominent during the later part of the protocol. Another possibility is that Ca^2+^ release is reduced with a delay in this model as observed in many isolated muscle fatigue protocols (Westerblad and Allen, 1991) and in the exteriorized mouse muscle (Figure 3B). The final example is from Allen et al. (2011) who studied the exteriorized tibialis anterior of mouse (Figure 2D). Blood flow was intact and the muscle temperature was 30°C. The protocol involved direct muscle stimulation with 400 ms tetani repeated every 4 s (duty cycle = 0.1). Force reached a plateau at ~50% after 6–10 min and P_i_ was determined in whole muscle extracts at the beginning and end of the protocol and after recovery (a typical fatigue protocol for this preparation is shown in Figure 3C). The three upper most points are control, recovery and the standard fatigue protocol. The two lowest points are a more intense stimulation protocol (duty cycle 0.2) and a protocol in which the muscle was ischaemic. There is a reasonable correlation over the whole range between force decline and P_i_ in this preparation.

All the results cited demonstrate a negative correlation between P_i_ and force during fatiguing stimulation. Typically the effect of P_i_ appears smaller early in fatigue and the effect appears to be enhanced in the steady state. A simple explanation of this would be if P_i_ also decreases Ca^2+^ release but after a delay.

## Measurements of P_i_ and Ca^2+^ in mouse muscle during fatigue

The data in Figure 1 make it clear that the expected reduction in force as a function of P_i_ is very dependent on the tetanic [Ca^2+^]_i_. Thus, to analyse the consequences of P_i_ during fatigue it is necessary to have measurements of force, tetanic [Ca^2+^]_i_ and P_i_. We have recently developed a new model of fatigue in which, potentially, all these data can be determined (Allen et al., 2011). The approach uses the tibialis anterior muscle of the anaesthetized mouse. The distal tendon of the muscle is detached and tied to a force transducer while the proximal end is undisturbed so that normal blood supply occurs. Ca^2+^ is measured using genetically-encoded cameleons (Miyawaki et al., 1997) transfected into the muscle 5–10 days before the experiment. The indicator is expressed in a subset of fibers and those within one or two fibers diameters of the outer surface of the muscle can be examined. Figure 3A shows a fluorescent XY image of the muscle surface obtained with a 2-photon microscope. In this image the scanning occurred from left to right so that at the left of the image a group of five fibers showing variable degrees of expression are visible. Approximately half way through the image acquisition (total time 730 ms) the muscle was stimulated tetanically, indicated by the movement artifact, and the right hand part of the image shows the same group of fibers during a tetanus. Cameleons have two fluorophores (YFP and CFP) attached to the ends of a calmodulin molecule plus a linker segment. When Ca^2+^ binds to calmodulin, it undergoes a shape change and the CFP and YFP become sufficiently close that fluorescence resonance energy transfer (FRET) occurs between them. The molecule is illuminated with a wavelength that excites CFP which then emits at its characteristic wavelength. In the presence of Ca^2+^, FRET occurs between CFP and YFP so that some fraction of the emitted light is now at the YFP emission wavelength. Thus, the ratio of YFP/CFP emitted fluorescence is Ca^2+^ sensitive.

Figure 3C illustrates our standard fatigue protocol. In the control period the muscle is stimulated with maximal tetani at 2 min intervals leading to stable tetanic force. During the fatiguing protocol the muscle is stimulated every 4 s (duty cycle = 0.1) for 12 min and force approaches a steady state by the end of this period. Subsequently the tetanic frequency is reduced so that at 2 min intervals tetani recover to around 90% of the initial force. Figure 3B shows the YFP/CFP ratios at rest and during selected tetani during the protocol. Resting [Ca^2+^]_i_ typically shows no significant change during this fatigue protocol. Tetanic [Ca^2+^]_i_ falls during fatigue and recovers afterwards. If the difference between tetanic and resting [Ca^2+^]_i_ is defined as 100%, then the average tetanic ratio during fatigue declined to 67%. The ratio then recovers with a timecourse similar to that of force. We have not calibrated our YFP/CFP ratios but data from Miyawaki et al. (1999) suggest that the relationship between YFP/CFP ratio and Ca^2+^ should be linear for our indicator (YC2) over the lower part of its range.

Metabolic measurements were made by rapidly freezing the muscle in liquid N_2_ and extraction with perchloric acid. P_i_ was measured in the supernatant using 31P-NMR spectroscopy. The relation between force and P_i_ in control (upper leftmost point) and various types of fatigue is depicted in Figure 2D and shows a robust linear relationship between P_i_ and force over the whole range of force outputs. In order to decide to what extent changes in P_i_ can explain the reduction in force one needs to compare data in Figure 2D with the data in Figure 1D. There are several problems involved in such a comparison. (1) One needs to know the appropriate level of Ca^2+^ in order to make the comparison. (2) Both the force and the metabolic measurements are averaged from the whole muscle which will consist of a mixture of fiber types. Percival et al. (2010) have determined the fiber type composition of mouse tibialis anterior and the main fiber types are Type IIB (54%) and Type IIA (34%) with smaller amounts of various intermediate fiber types. The Type IIB fibers predominate on the superficial half of the muscle while the Type IIA fibers are mainly located in the deeper portion. As a consequence, our Ca^2+^ measurements, which are made from superficial fibers, are likely to be from Type IIB fibers. Larsson et al. (1991) have determined the fatiguability of these fiber types in rat tibialis anterior using the single motor unit stimulation protocol. Using a duty cycle of 0.4, compared to our 0.1, Type IIB fibers fatigued to 2% control in 4 min whereas Type IIA fibers fatigued to 93% over 4 min. Assuming rat and mouse fibers are similar in fatiguability, then it would seem that Type IIA fibers should show very little fatigue at our duty cycle and, conversely, most of the observed fatigue in our preparation is likely to be in the Type IIB fibers.

**Figure 4:**
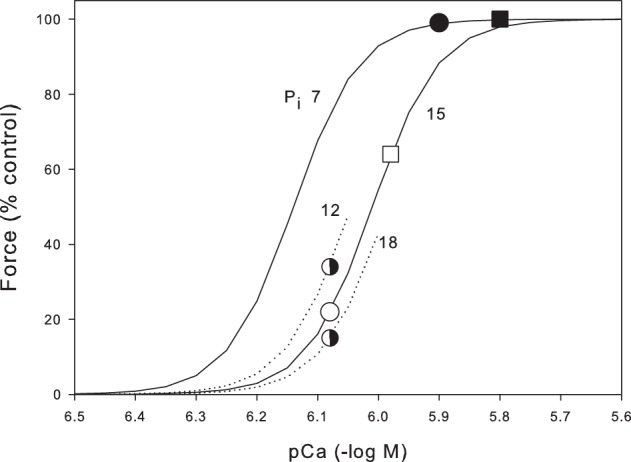
**Prediction of the force during fatigue based on the P_i_ and Ca^2+^ data from the mouse tibialis anterior.** Skinned fiber data (continuous lines) is from Debold et al. (2006) and shows pCa force data at 7 mM, the resting P_i_ in mouse tibialis anterior, and 15 mM, the P_i_ reached after the standard fatigue protocol shown in Figure 3. Black circle indicates the resting unfatigued muscle with P_i_ 7 mM and Ca^2+^ assumed just sufficient to fully saturate the contractile machinery (pCa 5.9). Open circle shows the predicted force when Ca^2+^ falls by 33%, as measured during fatigue, and P_i_ rises to that observed in fatigue. The skinned fiber data predict a force of 22% under these conditions. The closed and open square show another estimated with the Ca^2+^ higher by 25%. Also shown are parts of the pCa force curves for P_i_ 12 and 18 mM (see text for details).

To understand how P_i_ and Ca^2+^ interact during fatigue, in Figure 4 we plot skinned fiber data at 30°C (Debold et al., 2006) showing pCa/force curves at the resting P_i_ (7 mM) and the fatigued P_i_ (15 mM). Our Ca^2+^ measurements are not calibrated but, since we stimulate at a frequency that gives maximal force, we can assume that Ca^2+^ is at least supramaximal on the appropriate P_i_ curve (for recent review of calcium measurements in skeletal muscle see Baylor and Hollingworth, 2011). Thus, for the unfatigued muscle (P_i_ = 7 mM) reading from Figure 4, we can see that the tetanic [Ca^2+^]_i_ should be ~pCa = 5.9 giving a tetanic [Ca^2+^]_i_ = 1.26 μM. This point is marked on Figure 4 as a black circle. From our Ca^2+^ measurements we found that tetanic [Ca^2+^]_i_ reduced to 67% in the fatigued muscle giving a tetanic [Ca^2+^]_i_ of 0.83 μM (pCa 6.08). Simultaneously during fatigue P_i_ rises to 15 mM so the expected force for these conditions is given by the empty circle (22% control force). Thus, under these assumptions, the combination of Pi and [Ca^2+^]_i_ predict a force of 22% in our fatigue protocol whereas the measured fatigue force was 49%. This analysis assumes that both the fast and slow fibers had similar changes in Ca^2+^ and P_i_ and that the properties of the slow and fast fibers are similar with respect to P_i_. On the first point, Karatzaferi et al. (2001) measured metabolic changes in both Type IIA fibers and the human equivalent of Type IIB (Type IIXa). Although they did not measure P_i_, the decline in PCr was substantially greater in the IIXa fibers (103 mmol/kg dry weight) compared to the Type IIA fibers (71 mmol/kg dry weight). This suggests that P_i_ would be higher than the average in Type IIB fibers and correspondingly lower in the Type IIA fibers. On the second point Debold et al. (2006) state that Type IIB and IIA fibers had similar properties with respect to Ca^2+^ and P_i_ sensitivity. To further investigate this point we have recalculated the expected force assuming the muscle consists of 60% Type IIB and 40% Type IIA. We further assume that P_i_ is 20% greater in the Type IIB fibers (18 mM) and 20% less in the Type IIA fibers (12 mM). The half shaded circles (Figure 4) show that on these assumptions, the Type IIB fibers would generate 15% force while the Type IIA fibers would generate 34%. The whole muscle would then generate 23% (0.4 × 0.34 + 0.6 × 0.15). These new assumptions only change our estimated force from 22 to 23%. Thus, these two areas of uncertainty probably would not make a very substantial change in the estimated force under the fatigue conditions. On the other hand the estimated tetanic [Ca^2+^]_i_ makes a large contribution. For instance if we assume the tetanic [Ca^2+^]_i_ is 25% higher than required to saturate (black square in Figure 4), then the estimated force during fatigue after the appropriate reduction in tetanic [Ca^2+^]_i_ (open square) becomes 64%. Thus, a 25% change in our estimated Ca^2+^ leads to large (64 vs. 21%) estimate in the fatigued force. Furthermore, we have no information on the tetanic Ca^2+^ in Type IIA fibers or how it might change during fatigue. Our conclusion as present is that the combination of declining Ca^2+^ and rising P_i_ during fatigue are probably capable of explaining much of the observed force reduction. To make this estimate more precise we would need calibrated Ca^2+^ measurements in both fiber types and more details on the metabolite changes in different fiber types.

An unresolved difficulty with these calculations is to explain the observation of Larsson et al. (1991) showing that Type IIB fibers would be expected to fatigue to near 0 whereas Type IIA fibers show very little fatigue. In contrast our modeling suggests Type IIB fatigue to 15% while Type IIA fatigue to 34%. There are many possible causes of this discrepancy; one is that factors other than P_i_ and Ca^2+^ are important in fatigue. A second possibility is that the difference in P_i_ is greater between the two fiber types. A further possibility is that the Ca^2+^ changes occurring during fatigue are smaller in the Type IIA fibers and larger in the Type IIB fibers which would be expected if the Ca^2+^ changes are a function of the P_i_ changes as discussed earlier.

## Conclusions

Current data on the changes in P_i_ in muscle during fatigue suggest that the combination of the increase in P_i_ and the decrease in tetanic Ca^2+^ are capable of explaining much of the observed decline in force. This analysis is highly dependent on skinned fiber data obtained at 30°C and more data at 37°C would be desirable. Another area of uncertainty is the level of tetanic Ca^2+^ in the various fibers types and the extent to which this changes during fatigue protocols. While the mechanism of change in tetanic Ca^2+^ remains uncertain, a strong possibility is that the change in P_i_ is instrumental in the reduction of tetanic Ca^2+^ suggesting that evolution has utilized one metabolite to reduce both the myofibrillar performance and the Ca^2+^ release. The many other factors that modulate fatigue would appear to do so by fine-tuning the substantial reduction in force caused by the interactions between P_i_ and myofibrillar proteins and the Ca^2+^ release mechanism.

### Conflict of interest statement

The authors declare that the research was conducted in the absence of any commercial or financial relationships that could be construed as a potential conflict of interest.
